# Quality-adjusted life years for digital cognitive behavioural therapy for insomnia (Sleepio): a secondary analysis

**DOI:** 10.3399/BJGPO.2022.0090

**Published:** 2022-11-16

**Authors:** Elizabeth A Stokes, Richard Stott, Alasdair L Henry, Colin A Espie, Christopher B Miller

**Affiliations:** 1 Health Economics Research Centre, Nuffield Department of Population Health, University of Oxford, Oxford, UK; 2 National Institute for Health and Care Research Oxford Biomedical Research Centre, Oxford, UK; 3 Department of Psychiatry, University of Oxford, Oxford, UK; 4 Big Health Inc., CA, US and London, UK; 5 Sir Jules Thorn Sleep & Circadian Neuroscience Institute, Nuffield Department of Clinical Neurosciences, University of Oxford, Oxford, UK

**Keywords:** Insomnia; cognitive behavioural therapy, quality of life, quality-adjusted life years, sleep, health care economics and organisations

## Abstract

**Background:**

Insomnia is common, and difficulty with daytime functioning is a core symptom. Studies show cognitive behavioural therapy (CBT) improves functioning, but evidence is needed on its value for money. Quality-adjusted life years (QALYs), capturing length and quality of life, provide a standard metric by which to judge whether a treatment is worth its cost. Studies have found QALY gains with therapist-delivered and therapist-guided CBT, but most have not reached statistical significance. Estimates of QALY gains with fully automated digital CBT (dCBT) for insomnia are lacking.

**Aim:**

To assess whether dCBT (Sleepio) for insomnia is associated with gains in QALYs compared with a sleep hygiene education control.

**Design & setting:**

A secondary analysis of a large effectiveness trial of 1711 participants from the UK, US, and Australia.

**Method:**

EQ-5D scores, the National Institute for Health and Care Excellence's (NICE’s) preferred measure of health-related quality of life (HRQoL), were predicted (mapped) from the 10-item Patient-Reported Outcomes Measurement Information System (PROMIS-10) Global Health scores and used to determine QALYs from baseline to 24 weeks (controlled), and to 48 weeks (uncontrolled).

**Results:**

At week 24, QALYs were significantly higher for the dCBT group, with mean QALYs 0.375 and 0.362 in the dCBT and control groups, respectively. The mean difference was 0.014 (95% confidence interval [CI] = 0.008 to 0.019), and this difference was maintained over the 48-week study period (0.026, 95% CI = 0.016 to 0.036). The difference of 0.026 QALYs is equivalent to 9.5 days in perfect health.

**Conclusion:**

Sleepio is associated with statistically significant gains in QALYs over time compared with control. Findings may be used to power future studies and inform cost-effectiveness analyses of automated dCBT for insomnia scaled to a population level.

## How this fits in

Sleepio*,* a fully automated dCBT programme for insomnia, has been recommended as a clinically effective and cost-effective treatment by NICE. Previous studies have found gains in QALYs with therapist-delivered and therapist-guided CBT for insomnia, but in most reports these gains have not been found to be statistically significant. This is a concern as CBT is recommended as the first-line treatment. In this study, using data from a large effectiveness trial, it is found that Sleepio is associated with statistically significant gains in QALYs, equivalent to 9.5 days in perfect health.

## Introduction

Insomnia is common, with symptoms affecting up to 30% of the population.^1^ As a disorder, it is characterised by persistent difficulty initiating and/or maintaining sleep, or early-morning awakening with an inability to return to sleep.^2^ For a diagnosis to be made, individuals must also experience significant impairment to daytime functioning. Typical impacts are on mood, fatigue, concentration, or memory across different settings (for example, social and occupational). It has been recognised for some time that insomnia and its consequences negatively impact both physical and mental health dimensions of HRQoL.^3,4^ Indeed, real-world research suggests that insomnia is associated with a 10% reduction in HRQoL when compared with people who do not have insomnia.^4^

QALYs, calculated from HRQoL measures, are commonly used in economic evaluations of therapeutic interventions.^5^ A QALY is a composite measure of length and quality of life that can be used to compare outcomes across a broad range of disease areas and patient groups. They are used by the UK's NICE to evaluate whether a treatment represents good value for money.^6^ In short, QALYs therefore provide a standard metric by which to judge whether a treatment is worth the cost.

Previous studies have used QALYs to evaluate the cost-effectiveness of therapist-delivered and therapist-guided CBT for insomnia. Gains in QALYs have been observed but were not found to be statistically significant.^7,8^ This is a concern given that CBT is recommended as the first-line treatment for insomnia by the British Association of Psychopharmacology,^9^ and the American College of Physicians.^10^ Sleepio, a fully-automated dCBT-based treatment for insomnia, has been recommended by NICE as cost-saving^11^ by reducing primary care costs through fewer GP appointments and sleeping pills prescribed.^12^ Patients in England usually receive advice about sleep hygiene education (a set of behavioural recommendations to help promote better sleep^13^) or sleeping pills for their insomnia.^14,15^ NICE recommends a referral for face-to-face CBT for insomnia but has highlighted that this is not routinely available on the NHS for most people with insomnia and so recommends Sleepio because it costs less, is scalable to the population, and may be non-inferior to face-to-face CBT.^11^ Given that Sleepio has the potential to be a cost-effective treatment for insomnia,^16^ it is of interest to evaluate whether Sleepio is associated with statistically significant improvements in QALYs.

This study investigated potential gains in QALYs associated with Sleepio using HRQoL data from a published large effectiveness trial comparing Sleepio with a sleep hygiene control in 1711 participants.^17^ This Digital Insomnia therapy to Assist your Life as well as your Sleep (DIALS) trial observed improvements in insomnia, psychological wellbeing, and functional health, which were maintained at 48-weeks' follow-up.^18^ Using these data, individual participant responses were mapped from the PROMIS-10 Global Health scale to the EQ-5D. The EQ-5D is NICE’s preferred measure of HRQoL when calculating QALYs, and encompasses five dimensions of health (mobility, self-care, usual activities, pain and discomfort, and anxiety and depression). Its scores, known as utilities, are based on preferences, so how good or bad each health state is according to the general population (the value set). These utilities were then used to estimate QALYs to evaluate whether dCBT for insomnia resulted in gains to QALYs when compared with a sleep hygiene control.

## Method

First, the DIALS trial will be described, and then how QALYs were calculated.

### Trial design

This is a secondary analysis of the DIALS trial, a large effectiveness clinical trial of 1711 participants with insomnia recruited online from the UK, US, and Australia. Participants were recruited through online advertisements and contact lists where adults with insomnia volunteered to be involved in research and were randomised to either dCBT (*n* = 853) or sleep hygiene control (*n* = 858).^19^ Digital CBT was delivered using Sleepio, a fully automated dCBT programme comprising 6-weekly sessions containing cognitive, behavioural, and educational interventions. Content is delivered by an animated personal therapist, and algorithms drive treatment personalisation based on responses to questions and sleep diary data. The study assessed generic HRQoL in participants using PROMIS-10^20^ collected in the trial at baseline, 4 weeks, 8 weeks, 24 weeks (both groups), and an uncontrolled follow-up at 36 weeks and 48 weeks for the Sleepio group only.^18^ Items ask generally about overall health, quality of life, physical and mental health, social and physical activities, and fatigue. Items are scored 1–5, range: 10–50, with higher scores indicating better overall health.

### Calculating quality-adjusted life years

The EQ-5D is used to generate QALYs, and for this study, individual participant scores from the PROMIS-10 were first mapped to EQ-5D utilities using methods recommended by Thompson *et al*,^21^ building on previous work by Revicki *et al*.^22^ In other words, EQ-5D scores were predicted from PROMIS-10 Global Health scores. Eight items from the PROMIS-10 are used for mapping (general health, physical health, mental health, physical activities, pain [recoded], fatigue, social activities, and emotional problems). Specifically, the model applies equipercentile equating to the predicted values of a linear regression model, where PROMIS-10 items are treated as categorical predictors. Equipercentile equating translates scores from one scale to another by matching their cumulative distribution functions. The mapping by Thompson *et al* ^21^ uses the US value set for the EQ-5D-3L. The summary index scores (utilities) are then used to compute QALYs.

The QALY profile for each participant from baseline to 24 weeks was estimated, based on the EQ-5D scores, which range from 0 (dead) to 1 (perfect health), and their time points, and the area under the curve of utility measurements was used to calculate the number of QALYs accrued by each participant. QALYs were calculated assuming that each participant’s utility changes linearly between each of the time points (baseline, 4 weeks, 8 weeks, and 24 weeks). Beyond the controlled comparison to 24 weeks, assumptions were made about the control arm to extend analyses to 48 weeks using last observation carried forward. Missing EQ-5D data were first summarised descriptively, and exploratory analyses were conducted to understand possible mechanisms and patterns of missing data. Logistic regressions also explored associations between missingness and baseline variables, and missingness and previously observed EQ-5D scores. It was anticipated that multiple imputation would be required to impute missing values. Multiple imputation uses regression to predict *m* values for each missing data cell, based on key (complete and incomplete) variables. In line with guidance, multiple imputation using chained equations was conducted separately for each treatment group and the number of imputations, *m*, set to be at least equal to the percentage of incomplete cases.^23–25^

## Results

First, EQ-5D scores (mapped from the PROMIS-10) and QALYs from baseline to 24 weeks are presented, then from 24 weeks to 48 weeks, and finally they have been combined to estimate QALYs from baseline to 48 weeks.

### EQ-5D scores and QALYs to 24 weeks

#### Data completeness and handling of missing data

Overall, 43% of participants (*n* = 743/1711) had complete responses to the eight PROMIS-10 Global Health items used to map to the EQ-5D at each timepoint (baseline, 4 weeks, 8 weeks, and 24 weeks). Further details on EQ-5D scores and QALYs to 24 weeks based on observed data can be found in the supplementary material (see Supplementary Table S1). Exploratory analyses of missing data found, in line with the statistical analyses, a number of baseline variables that predict missingness, such as age and sex, and baseline variables that predict QALYs, including baseline EQ-5D. Findings support a missing at random assumption, thus multiple imputation is a flexible and appropriate method for handling the missing data.

Scores from the EQ-5D at 4 weeks, 8 weeks, and 24 weeks were imputed together with baseline EQ-5D, and other baseline variables: age, sex, country, partner status, employment status, smoking status, exercise status, history of heart disease, no comorbidities, other comorbidities, and number of comorbidities, separately for each treatment group (see Espie *et al* ^17^ for details of baseline variables). These baseline variables were included in the regression models since missingness may depend on them. Predictive mean matching with 10 nearest neighbours was used, so based on the variables included, the 10 most similar participants were identified, and the EQ-5D score for one randomly selected participant was assigned to the participant with missing data. The overall percentage of participants with any missing data was 57%: 61% in the dCBT arm and 52% in the control arm. Given this level of missing data, *m* = 61 imputations were conducted.

#### Results to 24 weeks

Table 1 shows EQ-5D scores and QALYs to 24 weeks in each group, with multiple imputation used to handle missing values. EQ-5D scores were higher in the dCBT arm at each time point following week 0, and were higher with statistical significance at week 8 and week 24. This results in significantly higher QALYs to 24 weeks in the dCBT arm. Note that the maximum number of QALYs that can be gained to 24 weeks by a participant is 0.460 (24 weeks x 7 days/365 days in a year).

**Table 1.** EQ-5D scores and QALYs to 24 weeks, with multiple imputation used to handle missing values

Outcome	dCBT (*n* = 853), mean (SE)	Control (*n* = 858), mean (SE)	Mean difference (95% CI)
**EQ-5D**			
Week 0	0.772 (0.004)	0.772 (0.004)	–0.0001(–0.012 to 0.012)
Week 4	0.799 (0.005)	0.785 (0.005)	0.014(–0.001 to 0.028)
Week 8	0.826 (0.005)	0.787 (0.005)	0.039(0.024 to 0.053)^a^
Week 24	0.820 (0.005)	0.787 (0.005)	0.033(0.018 to 0.048)^a^
**QALYs**			
0–24 weeks	0.375 (0.002)	0.362 (0.002)	0.014(0.008 to 0.019)^a^

^a^ *P*<0.05. dCBT = digital cognitive behavioural therapy. QALYs = quality-adjusted life years. SE = standard error.

### EQ-5D scores and QALYs from 24 weeks to 48 weeks

For the dCBT arm, EQ-5D scores at 24 weeks, 36 weeks, and 48 weeks were used to calculate QALYs as before. Supplementary Table S2 reports the EQ-5D scores and QALYs from 24 weeks to 48 weeks based on observed data. Missing data were imputed in line with methods described above. Given that the control group had access to dCBT from week 24, assumptions had to be made about the EQ-5D scores in the control arm. As mean EQ-5D scores in the control group were the same at week 8 and week 24 (see Table 1), it was assumed that individual EQ-5D scores at week 24 were carried forward to week 36 and week 48 for each participant. Table 2 reports the EQ-5D scores and QALYs from 24 weeks to 48 weeks, based on multiple imputation for the dCBT arm, and last observation carried forward for the control arm. This likely underestimates the variability in the control arm (and in the difference between groups). As for baseline to 24 weeks, EQ-5D scores and QALYs from 24 weeks to 48 weeks were significantly higher in the dCBT group.

**Table 2.** EQ-5D scores and QALYs from 24 weeks to 48 weeks, with multiple imputation used to handle missing values

Outcome	dCBT (*n* = 853), mean (SE)	Control (*n* = 858), mean (SE)	Mean difference (95% CI)
**EQ-5D**			
Week 24	0.820 (0.005)	0.787 (0.005)	0.033(0.018 to 0.048)^a^
Week 36	0.810 (0.006)	0.787 (0.005)	0.023(0.010 to 0.037)^a^
Week 48	0.817 (0.006)	0.787 (0.005)	0.030(0.016 to 0.043)^a^
**QALYs**			
24–48 weeks	0.375 (0.002)	0.362 (0.002)	0.013(0.007 to 0.018)^a^

^a^ *P*<0.05. dCBT = digital cognitive behavioural therapy. QALYs = quality-adjusted life years. SE = standard error.

### QALYs from baseline to 48 weeks

Finally, Table 3 combines the previous two analyses, to report QALYs from baseline to 48 weeks for both groups. Participants in the dCBT arm had significantly more QALYs overall than the assumed QALYs from participants in the control arm after 48 weeks. The difference of 0.026 QALYs is equivalent to 9.5 days in perfect health when extrapolated to 1 year. It is important to note that it was assumed EQ-5D scores, which were used to generate QALYs, were carried forward from the last controlled observation in the study at week 24 to the uncontrolled assessments at week 36 and week 48.

**Table 3.** Quality-adjusted life years from baseline to 48 weeks

QALYs	dCBT (*n* = 853), mean (SE)	Control (*n* = 858), mean (SE)	Mean difference (95% CI)
0 to 24 weeks	0.375 (0.002)	0.362 (0.002)	0.014(0.008 to 0.019)^a^
24 to 48 weeks	0.375 (0.002)	0.362 (0.002)	0.013(0.007 to 0.018)^a^
0 to 48 weeks	0.750 (0.004)	0.724 (0.003)	0.026(0.016 to 0.036)^a^

^a^ *P*<0.05. dCBT = digital cognitive behavioural therapy. QALYs = quality-adjusted life years. SE = standard error.

## Discussion

### Summary

Fully automated dCBT (Sleepio) for insomnia was associated with statistically significant improvements to QALYs relative to sleep hygiene control over 48 weeks. The difference of 0.026 QALYs is equivalent to 9.5 days in perfect health. Improvements in QALYs are likely owing to improved HRQoL with improved insomnia.

### Strengths and limitations

This study used patient data from a large and well-powered effectiveness trial of dCBT with a long follow-up duration. The mapping undertaken used equipercentile equating methods, which are preferred over regression-based methods since they avoid the issue of regression to the mean.^26^ PROMIS-10 items were treated as categorical predictors, not continuous predictors, which was a limitation of previous models. However, a limitation of this work is that the EQ-5D scores have been estimated from the PROMIS-10 questionnaire rather than measured directly. Furthermore, the mapping by Thompson *et al* ^21^ used the US value set for the EQ-5D, not the UK value set. In previous work, it has been suggested that the EQ-5D may not be sensitive enough to detect change in quality of life in response to improved symptoms of insomnia and more specific measures of mental health complaints may be considered in future work.^7^ Further research should now explore whether gains accrue over longer periods of time (>12 months) for both digital and therapist-delivered CBT compared with a control.

### Comparison with existing literature

Fully automated dCBT is an effective intervention for insomnia offering sustained benefits to functional health, psychological wellbeing, and sleep-related quality of life.^17,18^ This study extends previous findings and demonstrates that automated dCBT is also associated with statistically significant gains in QALYs when compared with a sleep hygiene control over 24 weeks. Gains in QALYs are likely from improved domains of HRQoL and were maintained over time when assessed under assumptions at 48-weeks' follow-up. The difference of 0.026 QALYs between groups is equivalent to 9.5 days in perfect health. Results appear to be the first to demonstrate statistically significant gains to QALYs with an automated dCBT intervention compared with a sleep hygiene control, the most common intervention used in general practice for insomnia management in the UK.^14,15^ Results may also be useful as they allow researchers to model the cost-effectiveness of dCBT for insomnia when delivered at a population level and compare results with other insomnia treatment options.

Insomnia is associated with reduced HRQoL for domains of physical, mental health, social, and emotional functioning.^3,4^ Successful insomnia treatment can improve functioning associated with HRQoL.^3^ Symptoms of insomnia, psychological wellbeing, and functional health (PROMIS-10) have all been found to improve with dCBT in the participants studied here.^17^ Findings suggest that gains to insomnia, wellbeing, and functional health with dCBT translate to gains in QALYs that are greater than gains from a sleep hygiene control. Results also show that both participants in the dCBT and control group had similar levels of estimated EQ-5D scores at baseline (0.772) and indicate moderate impairment in health status from insomnia disorder. By comparison, mean EQ-5D scores in the UK and US general populations are similar at 0.86 and 0.87, respectively.^27^ This impairment at baseline likely reflects reduced HRQoL found previously in those with insomnia.^3,4^ Previous studies have found gains in QALYs associated with therapist delivered^8^ and therapist-guided^7^ CBT for insomnia. Improvements to QALYs, however, were statistically significant in only one small study, which examined patients with insomnia and major depressive disorder and mapped QALYs from a depression rating scale.^28^

### Implications for research and practice

It is important to evaluate QALYs associated with dCBT because automated dCBT has the potential to provide access to CBT at a population scale in a cost-effective way. To date, widespread provision of recommended first-line CBT for insomnia has not been possible because of a lack of trained therapists. Patients are left with second-line sleep medication or ineffective sleep hygiene advice, which is counter to treatment guidelines.^9,10^ Digital delivery is more scalable and appears to be more cost-effective than therapist-delivered CBT as it provides similar benefit at a lower cost.^16^ Sleepio has previously demonstrated cost savings in UK primary care settings, reducing costs by approximately £70.44 per person.^12^ It is therefore likely to be more cost-effective compared with sleep hygiene advice and face-to-face CBT (estimated by NICE to cost £542 per person), if priced under £70 per person, as highlighted by NICE in their cost-saving recommendation for Sleepio.^11^ Subsequent research should now look to model cost-effectiveness from a societal perspective to further determine pricing. Results may be used to inform future studies that evaluate dCBT with therapist-delivered CBT and medications for insomnia with QALYs for cost-effectiveness in UK settings.^16^

## Trial registration number

ISRCTN: 60530898 — http://www.isrctn.com/ISRCTN60530898.
